# Phylogeographic dynamics and molecular characteristics of *Enterovirus* 71 in China

**DOI:** 10.3389/fmicb.2023.1182382

**Published:** 2023-05-19

**Authors:** Zi-Hui Ma, Amina Nawal Bahoussi, Pir Tariq Shah, Yan-Yan Guo, Li Dong, Changxin Wu, Li Xing

**Affiliations:** ^1^Institutes of Biomedical Sciences, Shanxi University, Taiyuan, China; ^2^Shanxi Provincial Key Laboratory of Medical Molecular Cell Biology, Shanxi University, Taiyuan, China; ^3^Shanxi Provincial Key Laboratory for Prevention and Treatment of Major Infectious Diseases, Taiyuan, China; ^4^The Key Laboratory of Chemical Biology and Molecular Engineering of Ministry of Education, Shanxi University, Taiyuan, China

**Keywords:** hand, foot and mouth disease (HFMD), phylogenetics, phylogeographic dynamics, recombination, amino acid variation, China, *Enteroviruses* 71 (EV71)

## Abstract

Enterovirus 71 (EV71) and coxsackievirus (CV-A16) are the major etiological agents of hand, foot and mouth disease (HFMD). This report reviewed the full-length genomic sequences of EV71 identified in different provinces of China between 1998 and 2019 (a total of 312) in addition to eight worldwide reference genomes to address the genomic evolution and genetic events. The main prevalent EV71 strians in China are C4 genotypes, co-circulating with a few A, B5, C1, and C2 subgenotypes. A new emerging subgenotype in China was identified and classified as B6 genotype. Phylogeographic analysis revealed multiple branches, where a Jiangsu strain 2006-52-9 (GenBank ID: KP266579.1) was linked to different subgenotypes through multiple long mutant branches, including the CV-A16 viruses through the A genotype. Furthermore, identification of 28 natural recombination events suggests that the emergence of new genotypes are associated with intratypic recombination involving EV71 strains and intertypic recombination between EV71 and CV-A16 strains. Compared with the structural proteins, the non-structural proteins of EV71 seem to be highly variable with the highest variable regions of peptidase C3 (3C protein), P2A, and the N-terminus of RNA-dependent RNA polymerase. This study updates the phylogenetic and phylogeographic information of EV71 and provides clues to the emergence of new genotypes of EV71 based on genetics.

## Introduction

Hand, foot, and mouth disease (HFMD) is a common acute viral infection that was first identified in New Zealand in 1957 (Seddon and Duff, 1971) and occurred primarily in children under 5 years old (Ishimaru et al., 1980). HFMD is caused by Enterovirus Group A, mainly the coxsackievirus A (CV-A16; Shinohara et al., 1999) and the *Enterovirus* A71 (EV-A71 or EV71; Yan et al., 2001; Wang et al., 2012) that is responsible for the most severe and fatal forms of HFMD.

*Enterovirus* 71 was originally isolated from the feces of a child with encephalitis in California, the United States in 1969 (Schmidt et al., 1974). EV71 infection is transmitted through fecal–oral route. The virus is ingested through contaminated hands, infected feces, blisters, saliva, or respiratory secretions (Wang and Liu, 2014). Although the clinical spectrum of most cases ranges from asymptomatic to mild disease with diarrhea, rash, and blistering lesions on hands, feet, and mouth mucous membranes, in recent years, EV71-caused HFMD led to more severe forms with myocarditis and disorders of the central nervous system (Lee, 2016; Yi et al., 2022).

The first two EV71 outbreaks occurred in the 1970s in Europe in Bulgaria and Hungary, respectively (Chumakov et al., 1979; Nagy et al., 1982). Since then, several outbreaks have been reported in Australia, Asia, America, and Europe, and currently, EV71 exists in most countries, predominantly in the Asia-Pacific region (Chang et al., 2016). Despite years of research, EV71 infection remains a big threat to human health, which requires better understanding of clear molecular and genetic characteristics.

*Enterovirus* 71, a non-enveloped virus of the genus *Enterovirus* in the *Picornaviridae* family, has a single-stranded positive RNA genome of approximately 7.4 kb in length (Yuan et al., 2018). The virion capsid comprises 60 envelope protein progenitors (Brown and Pallansch, 1995) with a single open reading frame (ORF) flanked by a 5′-untranslated region (UTR) and a 3′-UTR followed by a poly(A) tail. The ORF encodes a large polyprotein, processed into three precursor proteins P1, P2, and P3 during virus replication (Toyoda et al., 1986; Shen et al., 2008). P1 is a structural protein containing four capsid proteins (VP1–VP4; Shen et al., 2008). P2 and P3 encode non-structural proteins: (2A–2C) and (3A–3D), respectively (Huang et al., 2014). The VP1 capsid protein is the most external and the major antigenic determinant (Tee et al., 2010), crucial for EV71 identification and evolutionary genotyping. Based on VP1 nucleotide sequences, EV71 is divided into eight genotypes: A–H. Genotypes B and C are further subdivided into 14 sub-genogroups, B0–B7 and C1–C6 (Solomon et al., 2010; Chang et al., 2016). C1 and C2 are mainly identified in Europe and Asia Pacific (Van Tu et al., 2007; Huang et al., 2008; Solomon et al., 2010). C4 and B5 are the predominant subtypes in Taiwan (Wang et al., 2021), D and G are indigenous to India (Bessaud et al., 2014; Saxena et al., 2015), E is circulating in Africa (Fernandez-Garcia et al., 2018), and F in Madagascar (Bessaud et al., 2014). VP2 is also related to antigenicity and the virus-host interaction. VP2 is also involved in the virulence of EV71 through neddylation, a ubiquitin-like posttranslational modification by conjugating neural precursor cell-expressed developmentally downregulated protein 8 (NEDD8) to specific proteins to regulate biological activities. VP2 protein is neddylated at K69 residue to promote viral protein degradation and consequently suppress the virus’s multiplication (Wang et al., 2022).

The P2 and P3-derived proteins are responsible for EV71 replication and the modulation of host cells. The viral 2A^pro^ shuts down the protein synthesis machinery of the host cell to facilitate viral protein synthesis (Li et al., 2018), cleaves Melanoma Differentiation Associated gene 5(MDA5)-RIG-I-like receptors (RLRs) and the Mitochondrial antiviral-signaling protein (MAVS) at Gly209, Gly251, and Gly265 sites to prevent Pattern Recognition Receptors (PRRs) from recognizing the invading virus, inhibiting the activation of IRF3 (Interferon regulatory Factor 3), and suppressing IFN (interferon) production (Feng et al., 2014). 2A^pro^ cleaved NOD-like receptor thermal protein domain associated protein 3 (NLRP3) protein at G493-L494 and Q225-G226 sites, respectively, inhibiting its activation and promoting EV71 virus infection (Xiao et al., 2019). 2C^pro^ functions primarily as an NTPase (Guan et al., 2017), and is involved in the formation of viral replication complexes and counteracts the host-innate immunity through NF-κB signaling (Zheng et al., 2011; Du et al., 2015). 3A^pro^ is involved in protein–protein interactions through its N-terminus. 3C^pro^ promotes viral replication by inhibiting the innate immune system and causing apoptosis (Bai et al., 2020; Diarimalala et al., 2020; Wen et al., 2020). 3D^pro^, an RNA-dependent RNA polymerase (RdRp), is a crucial enzyme for viral replication (Wang et al., 2017) and is primarily responsible for synthesizing and extending the EV71 viral genome.

In Mainland China, HFMD was first reported in Shanghai in 1981, and the Human EV71 was first isolated during the HFMD 2007 outbreak in Linyi, Shandong Province (Zhang et al., 2009). The molecular epidemiology and phylogenetic analysis revealed the endemicity of C4 subgenotype in China (Zhang et al., 2009). Based on VP1 gene sequence analysis, C4 genotype comprises two subtypes, C4a and C4b, where C4b had been reported as the most prevalent in Mainland China before 2004, shifting later to C4a subtype (Zhang et al., 2009; Wang et al., 2021). After the 2007/2008 epidemics and outbreaks, the ministry of Health of China classified HFMD as a Category C notifiable disease on May 2, 2008.

Despite the attention given to HFMD by public health professionals and clinicians, the EV71 continues circulating in China, with an significant number of deaths registered among young children. From 2012 to the present, EV71 infection has emerged in various provinces, including Guangxi, Shandong, Chongqing, and Yunnan (Chen et al., 2017; Kou et al., 2020; Jia et al., 2021; Jiang et al., 2021). Differences in EV71 virulence and the absence of specific antiviral treatment against EV-A71 highlight the need to establish more effective surveillance systems and therapeutic agents against the EV71 pathogen. Due to the emergence and accumulation of new EV71 strain sequences in China, we systemically reviewed the genetic characteristics of circulating EV71 strains in China between 1998 and 2019 based on the publicly available complete EV71 genome sequences.

## Novel genotype of EV71 emerging in China

**Figure 1:**
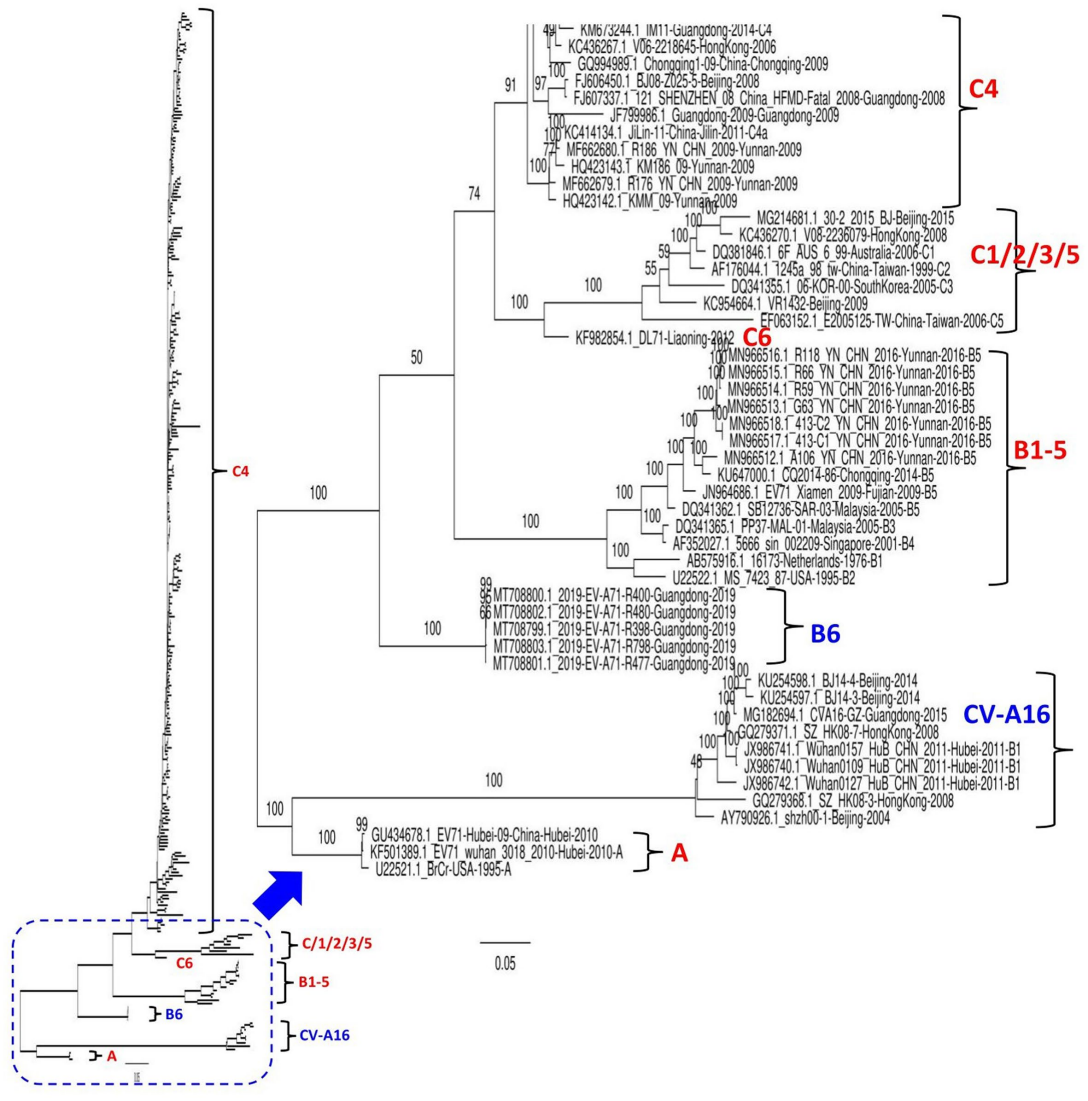
Maximum Likelihood (ML) phylogenetic tree based on 320 EV71 and CV-A16. The tree was constructed using the best-fit model GTR + F + G4 in IQ-TREE version 1.6.12 with 1,000 bootstraps. The percentage of trees is shown next to the branches. The viruses identified in China in this ML tree include nine CV-A16 strains and 303 EV-71 strains. The genotypes are as indicated. Eight extra viruses identified in other countries were included in this analysis as references, inluding 6F_AUS_6_99 (GenBank ID: DQ381846.1, Australia-2006, C1), 06-KOR-00 (GenBank ID: DQ341355.1, South Korea-2005, C3), 16173 (GenBank ID:AB575916.1, Netherlands-1976, B1), MS_7423_87 (GenBank ID: U22522.1, United States-1995, B2), PP37-MAL-01 (GenBank ID: DQ341365.1, Malaysia-2005, B3), 5666_sin_002209 (GenBank ID: AF352027.1, Singapore-2001, B4), SB12736-SAR-03 (GenBank ID: DQ341362.1, Malaysia-2005, B5), and BrCr (GenBank ID: U22521.1, USA-1995, A genotype). The viruses were identified as GenBank ID_virus name_country-province-year of collection. The detailed information about all viruses is seen in the Supplementary Figure S1.

A total of 320 EV71 complete sequences collected between 1998 and 2019, including all available 312 genomes from different provinces in China and eight worldwide reference genomes of different genotypes, were retrieved from the NCBI GenBank database. EV71 strains in the dataset were mostly from Beijing (*n* = 79), Yunnan (*n* = 42), Guangdong (*n* = 39), Shanghai (*n* = 26), and Zhejiang (*n* = 23; Supplementary Table S1). The nucleotide sequences were aligned using ClustalW in the BioEdit version 7.2.5 package. The maximum likelihood (ML) phylogenetic tree based on the full-length genome sequence was constructed using the best-fit model GTR + F + G4 in IQ-TREE version 1.6.12 (Trifinopoulos et al., 2016) with 1,000 bootstrap replicates. The common average length of all involved genomic strains was ~7,428 nt. As indicated in Figure 1 and Supplementary Figure S1, our phylogenetic tree based on the whole genome sequences classified EV71 into two main clusters, the first branch clusters CV-A16 and EV71-A genotype strains; meanwhile, the second branch clusters strains of the EV71-B and C genotypes. The results revealed that the quasi-totality of EV71 strains circulating in China between 1998 and 2019 cluster into genotype C4, and a few others (33 strains) cluster into genotypes (A, C1, C2, C5, C6, B5, and B6). The virus DL71 (GenBank ID: KF982854.1) identified in Liaoning in 2012 is genetically far distant from the viruses of C1, C2, C3, and C5, therefore designated as a new genotype of C6 (Liu et al., 2022). Notably, China EV71 strains circulating in 2019, all collected in Guangdong province (GenBank ID: from MT708799-MT708803), clustered independently into a separate new subgroup assigned as a novel genotype B6, implying the continuous emergence of new EV71 variants. These five strains were previously reported as belonging to C1 genotype on NCBI genbank database (Lv and Zhang, 2022); however, our phylogenetic analysis found them highly divergent from the C1 genotype. The B5 genotype included seven strains from Yunnan and one from each of Fujian and Chongqing. The CV-A16 strains (previously referred to as B1) clustered together into one independent clade encompassing nine strains from Beijing (3), Guangdong (1), Hong Kong (2), and Hubei (3; Figure 1; Supplementary Figure S1).

To increase the stringency of our phylogenetic tree results, a similarity analysis was performed to compare the genome of SH-17/SH/CHN/2002 (GenBank ID: JX678885.1, C4) strain to seven representative EV71 full-length sequences from A, B5, B6, C1, C4, and CV-A16 using SimPlot analysis (Lole et al., 1999). As shown in Supplementary Figure S2, KM186/09 (GenBank ID: HQ423143.1, C4) and BJ08-Z025-5 (GenBank ID: FJ606450.1, C4) strains showed the greatest similarity levels at P1, P2, and P3 (3A-3B-3C) coding regions (≥95%), and P3 (3D) region (≥75%) followed by 2019-EV-A71-R400 (GenBank ID: MT708800, B6) and 30-2/2015/BJ (GenBank ID: MG214681.1, C1) that revealed similarity levels ≥80% at P1 and P2 (2A) coding regions and ~ 60% at the P2 (2B-2C) and P3 coding regions. However, Beijing strain 30-2/2015/BJ is more similar to KM186/09 and BJ08-Z025-5 at the P3 (3D) coding region than to 2019-EV-A71-R400 (GenBank ID: MT708800, B6). CV-A16 Wuhan0127/HuB/ CHN/2011 (GenBank ID: JX986742) revealed the lowest sequence similarity (~ 25%) at the structural P1 coding region and ~ 50% at the nonstructural P2 and P3 coding regions (Supplementary Figure S2). Therefore, these findings show that EV71 representative strains fall into distinct groups, corroborating our phylogenetic tree results and suggesting that the defined EV71 genotypes are distinctly, highly specific, and divergent.

## Phylogeographic network of the full-length genome of EV71 in China

**Figure 2:**
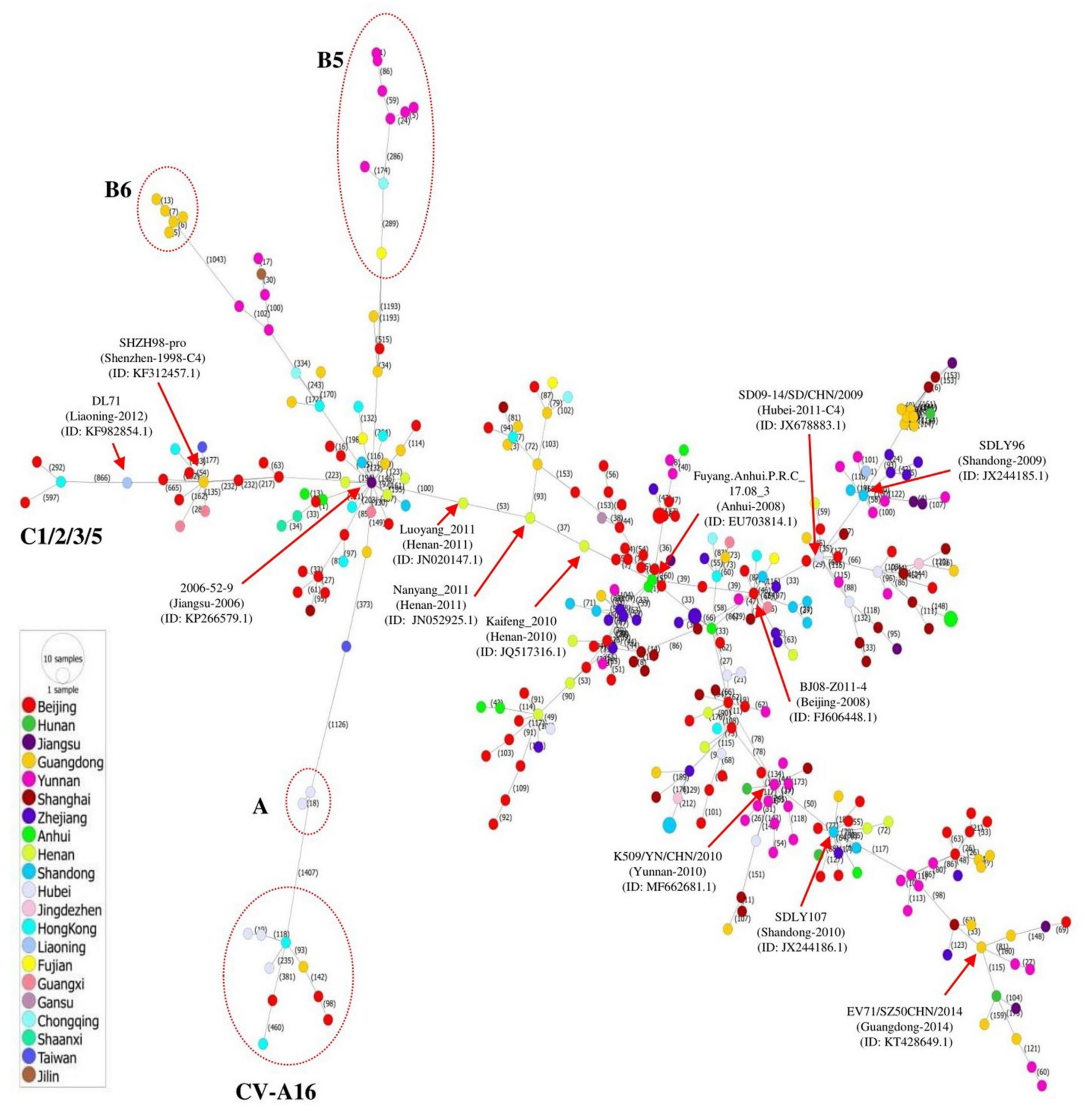
Phylogeographic network analysis of 312 full-length EV71 sequences collected in China between 1998 and 2019. The phylogenetic network was inferred using the MSN network implemented by PopArt v1.7. The C4 strains dominated the network, and formed several major branches. Genotypes A, B5, B6, and C1/2/3/5 were linked to virus 2006-52-9 (GenBank ID: KP266579.1) identified in Jiangsu province of China in 2006. The A genotype also gave rise to the CV-A16 isolates by long mutation branches. The viruses identified in different provinces of China are indicated with different colors.

The transmission of EV71 in China at the regional level was visualized by constructing a phylogeographic network of whole genome sequences of EV71 and several CV-A16 strains that was defined as B1. A haplotype network was constructed using the Minimum Spinning Network (MSN) method implemented by PopArt v1.7 (Leigh and Bryant, 2015) and showed that EV71 distribution was highly diverse, containing a major network Cluster with multiple mutant sub-branches (Figure 2). The strains isolated in Jiangsu, Anhui, Beijing, and Hubei showed a great diversity, expressed in most sub-branches, consistent with the phylogenetic tree. Among the multiple mutant branches, the virus 2006-52-9 (GenBank ID: KP266579.1) identified in Jiangsu province of China in 2006 linked to all other sub-genotypes including B5, B6, A, and C1/2/3/5 via long mutant branches, and to CV-A16 strains by a long mutant step on the A branch. The 2008 outbreak strain in Anhui (Fuyang.Anhui.P.R.C_17.08_3, GenBank ID: EU703814.1) was linked to multiple strains by short mutant branches, spreading mainly in Beijing and Jiangsu. Another major long branch is the aggregation of strains from Yunnan, Shandong, and Guangdong, with multiple short mutant branches (Figure 2).

## Naturally occurring inter- and intra-type recombinations of EV71

To explore the underlying mechanisms and assess the relative contribution of recombination to the high genetic heterogeneity and dissimilarity observed among EV71, we performed a recombination analysis of 312 EV71 full-length genomes (~7,428 nt) using a series of seven algorithms (RDP, Geneconv, BootScan, MaxChi, Chimera, SiScan, and 3Seq) implemented in the recombination detection program 4 (RDP4; Martin et al., 2015). A sequence was considered a potential recombinant if at least four of seven assays showed significant differences. Twenty-eight potential natural recombination events were detected in China between 1998 and 2019 (Supplementary Table S2), mainly intratypic and occurring between C4 strains (22 Events), whereas only six were intertypic (Events 1, 2, 4, 7, 27, and 28).

Interestingly, The newly identified B6 genotype seems to emerge by genomic recombination as shown in Event 27: 2019-EV-A71-R398 (GenBank ID: MT708799), resulting from genetic exchanges between EV71-Hubei-09-China (GenBank ID: GU434678, A genotype) and R186/YN/CHN/2009 (GenBank ID: MF662680.1, C4 genotype) as minor and major parental sequences, respectively (Supplementary Table S2). Our analysis identified another strain from the same B6 genotype: 2019-EV-A71-R798 (GenBank ID: MT708803.1), involved in the recombination as a major parental sequence (Event 28), confirming the contribution of recombination to the emergence of new variants of EV71.

Furthermore, intertypic recombination between EV71 and CV-A16 strains was detected involving two CV-A16 strains: BJ14-4 (GenBank ID: KU25459) in Events 2 and 4, and CVA16-GZ-Guangdong (GenBank ID: G182694.1) in Event 7. To our knowledge, the analysis identified for the first time natural genetic exchanges between EV71 strains in Event 7 to generate a CV-A16 strain CVA16-GZ (Supplementary Table S2).

Also, several strains were identified simultaneously involved in multiple recombination events (Supplementary Figure S3). For example, the strain DL71 (GenBank ID: KF982854.1) calssified as C6 was resulted from and involved in nine recombination events (Events 6, 8, 12, 13, 14, 15, 18, 22, and 28, respectively). Strain SHZH98 (GenBank ID: AF302996.1) was involved in eight recombination events (Events 5, 6, 8, 17, 18, 22, 26, and 28, respectively).

The breakpoints of the 28 identified recombinants are shown in Supplementary Figure S3, and that of nine recombinants were located, mainly at the 5′ and 3′ ends, among which six events occurred near the 5′-UTR (Events 2, 15, 16, 23, 24, and 25) and three near the 3′-UTR (Events in 3, 23, and 24). Events (2,4,7) occurred all over the P1 polyprotein, while Events (6, 10, 11, 18) occurred within VP3, Event 28 within the VP4, and Events () within the VP2 domain. Four Events were mapped at the overlap region: P2 (2C)-P3 (Events 1, 4, 8, and 21), and Events (9, 12, 14, 20, and 27) were within the P3 polyprotein, suggesting these regions to be hot spots for recombination. The recombination events were further confirmed by phylogenetic trees based on two different genomic regions (nt 1,200–2,400 and 4,500–5,500; Supplementary Figure S4), where the recombinant strain (GenBank ID: HM807310.1) in event one was genetically closer to the major parent V05-2243055 (GenBank ID: KC436266.1) in the nt 1,200–2,400 based phylogenetic tree (Supplementary Figure S4A) and became closer to the minor parent EV71/wuhan/3018/2010 (GenBank ID: KF501389.1) in the nt 4,500–5,500 based phylogenetic tree. In event 2, the recombinant strain (GenBank ID: KC954664.1) was genetically closer to the major parent BJ14-4 (GenBank ID: KU254598.1) in the nt 4,500–5,500 based phylogenetic tree; however, it turned out to be closer to the minor parent BJ110 (GenBank ID: HM002486.1) in the nt 1,200–2,400 based phylogenetic tree. Similar results were found for the remaining recombination events. These results supported the recombination analysis findings, indicating that the detected recombination is derived from real natural events.

## Amino acids variability landscape of EV71 polyprotein

Another genetic characteristic of EV71 is the amino acid variability of viral polyprotein, which was assessed by using Wu-Kabat variability coefficient (Garcia-Boronat et al., 2008) and 312 full-length protein sequences decuced from coding sequences of EV71 identified in China (Supplementary Table S1). The polyprotein contains a total of 2,190 amino acids, splits into structural proteins VP1-VP4 (P1 region) and non-structural proteins (P2 and P3 regions) during viral replication. The results showed a large variability in all regions with the highest variable regions encoding for non-structural 3C protein (also known as Peptidase C3, aa 1,639–1,643, highest recorded value of 9.5; Supplementary Figure S5C), followed by aa 910–960 in the P2A (Supplementary Figure S5B) and aa 1,730–1,875 in the N-terminus of RdRp (Supplementary Figure S5C). The structural proteins including VP1-VP4 are relatively conserved (Supplementary Figures S5A,B).

## Concluding remarks

In summary, the present study found that China EV71 strains are characterized by high recombination levels. The 28 identified natural recombinants involved C4 genotypes, suggesting that the EV71-C4 genotype remains the major threat to HFMD in China. It has been reported that CV-A16 circulating in China are recombinant viruses with unknown exact parental strains (Zhao et al., 2011). Our analysis determined recombination between EV71 and CV-A16, where the same CV-A16 strain (GenBank ID: KU254598.1) was involved in two recombinations with different EV71 C4 genotypes. Furthermore, an A-genotype strain (GenBank ID: KF501389.1) was determined to be involved in recombination with different C4 genotype strains to produce C4 subgenotypes of EV71 and CV-A16, respectively, indicating that recombination between different subgenotypes can produce new genotypes. The recombination not only drove the expansion of the EV71 genotype but also was a key factor in facilitating the emergence of new evolutionary lineages, which added to the complexity and heterogeneity of the EV71 disease epidemic in China additional challenges for the development of effective prevention strategies.

## Data availability statement

The original contributions presented in the study are included in the article/Supplementary material; further inquiries can be directed to the corresponding author.

## Ethics statement

For this retrospective type of study, formal consent is not required. Statement on the welfare of animals is not applicable as sample collection from animals has been done before.

## Author contributions

LX and Z-HM: conceptualization. PT, Z-HM, Y-YG, and AN: data analysis. PT: visualization. AN and Z-HM: writing. CW and LX: administration. AN, Z-HM, LD, and LX: manuscript revision. All authors contributed to the article and approved the submitted version.

## Funding

This work was supported by the Program of Introducing Talents of Discipline to Universities (D21004). Research Project was supported by Health Commission of Shanxi Province (2021XM20) and Key Research and development program of Shanxi Province (No. 202102130501009) for LD.

## Conflict of interest

The authors declare that the research was conducted in the absence of any commercial or financial relationships that could be construed as a potential conflict of interest.

## Publisher’s note

All claims expressed in this article are solely those of the authors and do not necessarily represent those of their affiliated organizations, or those of the publisher, the editors and the reviewers. Any product that may be evaluated in this article, or claim that may be made by its manufacturer, is not guaranteed or endorsed by the publisher.
