# Supplementary material for: Phylogeographic dynamics and molecular characteristics of Enterovirus 71 in China
Source: Front Microbiol. 2023 May 19;14:1182382. doi: 10.3389/fmicb.2023.1182382 (PMC10235518; doi:10.3389/fmicb.2023.1182382)
Supplement: Supplementary file 1 [file Data_Sheet_1.PDF]

Supplementary Table S1. Geographical distribution of EV71 genotypes and sub-genotypes in China during 1998-2019.

**EV71 genotype**	**C4**	**C1**	**C2**	**C5**	**C6**	**B6**	**B5**	**A**	**B1 (CV-A16)**	Total
Guangdong	33					5			1	39
Yunnan	34	1					7			42
Chongqing	3						1			4
Fujian	3						1			4
Hubei	7							2	3	12
Hongkong	10		1						2	13
Beijing	73	2	1						3	79
Hunan	4									4
Jiangsu	8									8
Shanghai	26									26
Zhejiang	23									23
Anhui	12									12
Shandong	14									14
Henan	16									16
Jiangxi	1									1
Guangxi	5									5
Gansu	1									1
Shaanxi	2									2
Taiwan	2		1	1						4
Jilin	1									1
Liaoning	1				1					2
										312

Supplementary Table S2. Identification of 28 potential recombination events in the genome of EV71 in China. The potential recombination events were analyzed using each of 7 algorithms (RDP, GENECONV, Bootscan, MaxChi, Chimaera, SiScan, and 3seq) in the RDP4 software. Genogroups were defined based on this study's full-length genomes-based phylogenetic tree (see Figure 1).

Event serial NO	Recombinant		Minor parent		Major parent		Detection methods						
	GenBank ID: Virus name (Province-Year)	Geno-group	GenBank ID: Virus name (Province-Year)	Geno-group	GenBank ID: Virus name (Province-Year)	Geno-group	R	G	B	M	C	S	T
1	HM807310.1:cmuh-050530-5 (Taiwan-2005)	C4	**KF501389.1:EV71/wuhan /3018/2010(Hubei-2010)**	A	KC436266.1:V05-2243055 (Hong Kong-2005)	C4	+	+	+	+	+	+	+
2	KC954664.1:VR1432 (Beijing-2009)	C4	HM002486.1:BJ110(Beijin g-2008)	C4	**KU254598.1:BJ14-4 (Beijing-2014)**	**(CV-A16)**	+	+	+	+	+	+	+
3	*MF662680.1:R186/YN/CHN/2 009 (Yunnan-2009)	C4	KP289419.1:EV71/P1034/2 013/China (Beijing-2013)	C4	HQ423143.1:KM186/09 (Yunnan-2009)	C4	+	+	+	+	+	+	+
4	*KC436270.1:V08-2236079 (Hong Kong-2008)	C4	JX678885.1:SH-17/SH/CHN/2002 (China-Beijing-2002)	C4	**KU254598.1:BJ14-4 (Beijing-2014)**	**(CV-A16)**	+	+	+	+	+	+	+
5	*JF799986.1:Guangdong 2009 (Guangdong-2009)	C4	KP266579.1:2006-52-9 (Jiangsu-2006)	C4	AF302996.1:SHZH98 (Beijing-2000)	C4	+	+	+	+	+	+	+
6	*AF302996.1:SHZH98 (Beijing-2000)	C4	KF982854.1:DL71(Liaonin g-2012)apiens)	C6	KF312457.1:SHZH98 pro (Shenzhen-1998)	C4	+	+	+	+	+	+	+
7	*** G182694.1_CVA16-GZ-Guangdong (Guangdong-2015)**	**(CV-A16)**	GQ994992.1:Henan2-09-China (Henan-2009)	C4	**KF501389.1:EV71/wuhan/3 018/2010 (Hubei-2010)**	A	+	+	+	+	+	+	+
8	KF982854.1:DL71 (Liaoning-2012)	C6	AF302996.1:SHZH98(Beiji ng-2000)	C4	MG214681.1:30-2/2015/BJ (Beijing-2015)	C4	+	+	+	+	+	+	+
9	KP289419.1:EV71/P1034/2013/ China (Beijing-2013)	C4	KC436265.1:V04-2218217(Hong Kong-2004)	C4	KY582572.1:EV71-MZ (Guangdong-2015)	C4	+	-	+	+	+	+	+
10	KP198624.1:Henan-ZMD/CHN/2012 (Hubei-2012)	C4	KP198623.1:Hubei-WH/CHN/2012 (Hubei-2012)	C4	MF662682.1:R262/YN/CHN/ 2010 (Yunnan-2010)	C4	+	+	+	+	+	+	+
11	KP289432.1:EV71/P990/2013/C hina (Beijing-2013)	C4	KP289430.1:EV71/P868/20 13/China(Beijing-2013)	C4	KP289427.1:EV71/P454/201 3/China (Beijing-2013	C4	+	+	+	+	+	+	+
12	*KF982854.1:DL71 (Liaoning-2012)	C6	JX678886.1:SH-6/SH/CHN/2002 (Beijing-2002)	C4	KC436270.1:V08-2236079 (Hong Kong-2008)	C4	+	+	+	+	+	+	+
13	*KF982854.1:DL71 (Liaoning-	C6	JX678874.1:CQ03-	C4	MG214681.1:30-2/2015/BJ	C4	+	+	+	+	+	+	+

	2012)		1/CQ/CHN/2003 (Beijing-2003)		(Beijing-2015)								
14	*KF982854.1:DL71 (Liaoning-2012)	C6	MF662680.1:R186/YN/CHN/2009 (Yunnan-2009)	C4	MG214681.1:30-2/2015/BJ (Beijing-2015)	C4	+	+	+	+	+	+	+
15	*KF982854.1:DL71 (Liaoning-2012)	C6	GQ994989.1:Chongqing1-09-China (Chongqing-2009)	C4	KC436270.1:V08-2236079 (Hong Kong-2008)	C4	+	+	+	+	+	+	+
16	MF662700.1:K38/YN/CHN/2013 (Yunnan-2013)	C4	MF662701.1:R50/YN.CHN/2013 (Yunnan-2013)	C4	MF662699.1:K34/YN/CHN/2013 (Yunnan-2013)	C4	+	+	+	+	+	+	+
17	AF302996.1:SHZH98 (Beijing-2000)	C4	EU703812.1:EV71/Fuyang.Anhui.P.R.C/17.08/1 (Anhui-2008)	C4	KF312457.1:SHZH98 pro (Shenzhen-1998)	C4	+	+	+	+	+	+	+
18	*KF982854.1:DL71 (Liaoning-2012)	C6	KY014080.1:839 (Zhejiang-2008)	C4	AF302996.1:SHZH98 (Beijing-2000)	C4	+	+	+	-	+	+	+
19	KT345959.1:120/EV71/Wenzhou/CHN/2014 (Zhejiang-2014-C4a)	C4	KU936120.1:SHAPHC5218/SH/CHN/14 (Shanghai-2014-C4a)	C4	KP198623.1:Hubei-WH/CHN/2012 (Hubei-2012)	C4	+	+	+	+	+	+	+
20	MF662680.1:R186/YN/CHN/2009 (Yunnan-2009)	C4	KP289417.1:EV71/P1027/2013/China (Beijing-2013)	C4	KM673244.1:IM11 (Guangdong-2014)	C4	+	-	+	+	+	+	+
21	*FJ606450.1:BJ08-Z025-5 (Beijing-2008)	C4	KJ004554.1:EV71/Hun12-14/2012 (Beijing-2010)	C4	JF799986.1:Guangdong 2009 (Guangdong-2009)	C4	+	+	+	+	+	+	+
22	*KF982854.1:DL71 (Liaoning-2012)	C6	AF302996.1:SHZH98 (Beijing-2000)	C4	KP289432.1:EV71/P990/2013/China (Beijing-2013)	C4	+	+	+	-	-	+	+
23	*MF662691.1:R6/YN/CHN/2011 (Yunnan-2011)	C4	KF142412.1:HNCZ/201208 (Hunan-2013)	C4	MF662683.1:R464/YN/CHN/2010 (Yunnan-2010)	C4	+	+	+	+	+	+	+
24	*KF142412.1:HNCZ/201208 (Hunan-2013)	C4	KF142411.1:HNCZ/201203 (Hunan-2013)	C4	MF662683.1:R464/YN/CHN/2010 (Yunnan-2010)	C4	+	+	-	+	+	+	+
25	*MF662685.1:R642/YN/CHN/2010 (Yunnan-2010)	C4	KP266579.1:2006-52-9 (Jiangsu-2006)	C4	MF662686.1:R993/YN/CHN/2010 (Yunnan-2010)	C4	+	+	+	-	-	-	+
26	*KP289432.1:EV71/P990/2013/China (Beijing-2013)	C4	AF302996.1:SHZH98 (Beijing-2000)	C4	JF894382.1:MP9-2 (Beijing-2011)	C4	+	-	+	+	+	+	+
27	MT708799.1:2019-EV-A71-R398 (Guangdong-2019)	B6	**GU434678.1:EV71-Hubei-09-China (Hubei-2010)**	**A**	MF662680.1:R186/YN/CHN/2009 (Yunnan-2009)	C4	+	-	-	+	+	+	+
28	*KF982854.1:DL71(Liaoning-2012)	C6	AF302996.1:SHZH98 (Beijing-2000)	C4	MT708803.1:2019-EV-A71-R798 (Guangdong-2019)	B6	+	+	+	+	+	+	-

R, RDP; G, GENECONV; B, BootScan; M, MaxChi; C, Chimaera; S, SiScan; T, 3Seq. +, verified; -, not verified.
* The major or minor parent may be the actual recombinant due to the possibility of misidentification.

Supplementary Table S3. The EV71 isolated in China and involved in multiple recombination events in the full-length genome.

GenBank ID: Virus name (province-year)	Event serial NO	GenBank ID: Virus name (Province-year)	Event serial NO
KF982854.1:DL71(Liaoning-2012)	Event 6, 8, 12, 13, 14, 15, 18, 22, 28	KP266579.1:2006-52-9 (Jiangsu-2006)	Event 5, 25
AF302996.1:SHZH98 (Beijing-2000)	Event 5, 6, 8, 17, 18, 22, 26, 28	KF312457.1:SHZH98 pro (Shenzhen-1998)	Event 6, 17
MF662680.1:R186/YN (Yunnan-2009)	Event 3, 14, 20, 27	MG214681.1:30-2/2015/BJ (Beijing-2015)	Event 8, 13, 14
KP289432.1:EV71/P990(Beijing-2013)	Event 11, 22, 26	KP198623.1:Hubei-WH/CHN/2012 (Hubei-2012)	Event 10, 19
KF501389.1:EV71/wuhan/3018 (Hubei-2010)	Event 1, 7	KC436270.1:V08-2236079 (HongKong-2008)	Event 12, 15
KU254598.1:BJ14-4(Beijing-2014)	Event 2, 4	MG214681.1:30-2/2015/BJ (Beijing-2015)	Event 13, 14
JF799986.1: Guangdong-2009 (Guangdong-2009)	Event 5, 21	KF142412.1:HNCZ/201208 (Hunan-2013)	Event 23, 24
MF662683.1:R464/YN/CHN/2010 (Yunnan-2010)	Event 23, 24		
