# Supplementary material for: Phylogeographic dynamics and molecular characteristics of Enterovirus 71 in China
Source: Front Microbiol. 2023 May 19;14:1182382. doi: 10.3389/fmicb.2023.1182382 (PMC10235518; doi:10.3389/fmicb.2023.1182382)

Supplementary Figure S1. The full ML phylogenetic tree based on 320 EV71 and CV-A16. The tree was constructed using the best-fit model GTR+F+G4 in IQ-TREE version 1.6.12 with 1000 bootstraps. The percentage of trees is shown next to the branches. The viruses were identified as GenBank ID_virus name_country-province-year of collection.

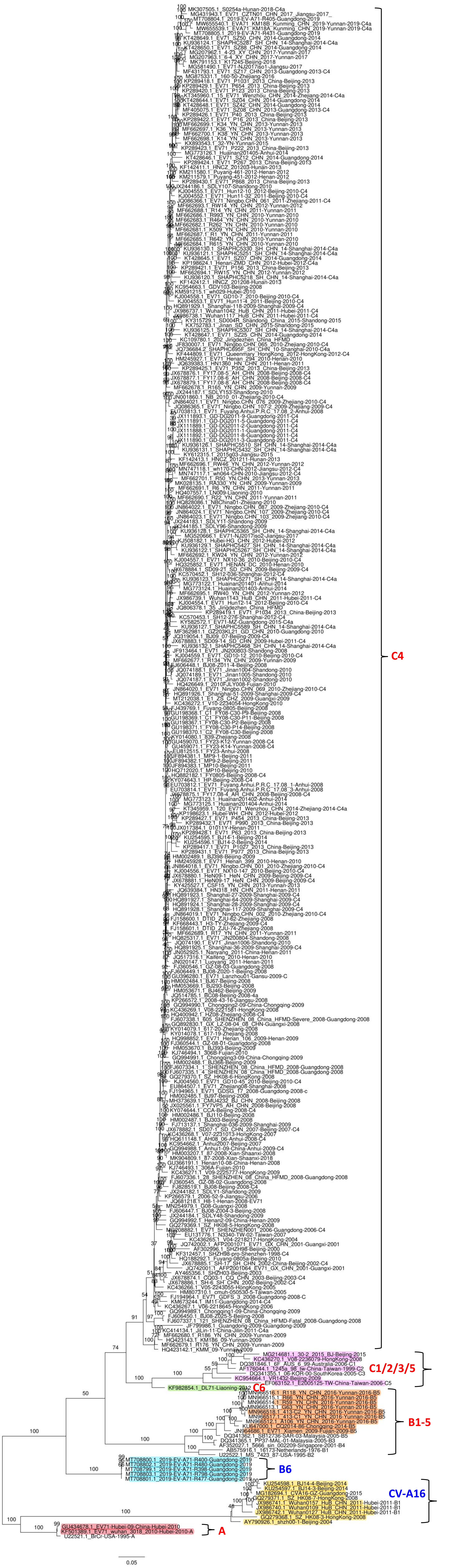

Supplementary Figure S2. Genomic similarity analysis of representative viruses selected from each genotypes. (**A**) Linear genomic structure of the EV71 including the polyprotein ORF. (**B**) Seven representative EV71 strains from each genotype in the phylogenetic tree of Figure 1 were compared using genomic similarity plot carried out with SimPlot ver.3.5.1. The X-axes show the nucleotide similarity percentage, and the Y-axes show the nucleotide position. The similarity is presented in percentage (%). The genome of virus SH-17/SH/CHN/2002 (GenBank ID: JX678885.1; China-Beijing 2002, C4) was used as SimPlot Query.

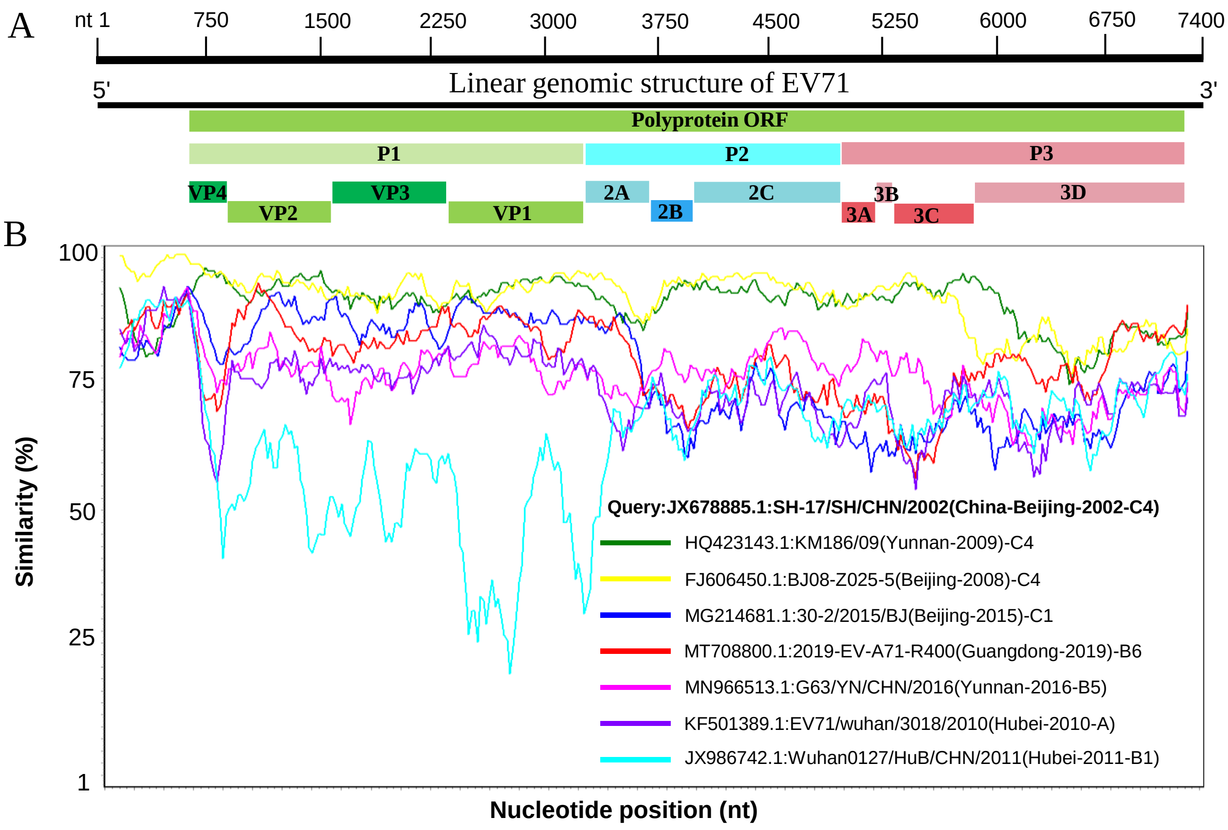

Supplementary Figure S3. Genetic recombination analysis of 312 full-length EV71 genomes. (**A**) Diagram showing the full-length genome of EV71 and the corresponding regions encoding P1, P2 and P3 domains. The P1 gene encodes four subdomains: VP4, VP2, VP3, and VP1; P2 gene encodes three subdomains: 2A, 2B, and 2C; P3 gene encodes four subdomains: 3A, 3B, 3C, and 3D. (**B**) Schematic representation of the potential recombination events listed in Supplementary Table S2. Recombination event serial numbers and the description of potential recombinants (GenBank ID: virus name/country-province-year of collection) are shown on the left. The pink and green blocks represent the DNA regions from minor or major parent viruses, respectively. The numbers on the top of filled green blocks indicate the nucleotide positions of breakpoints relative to the corresponding recombinant viruses on the left.

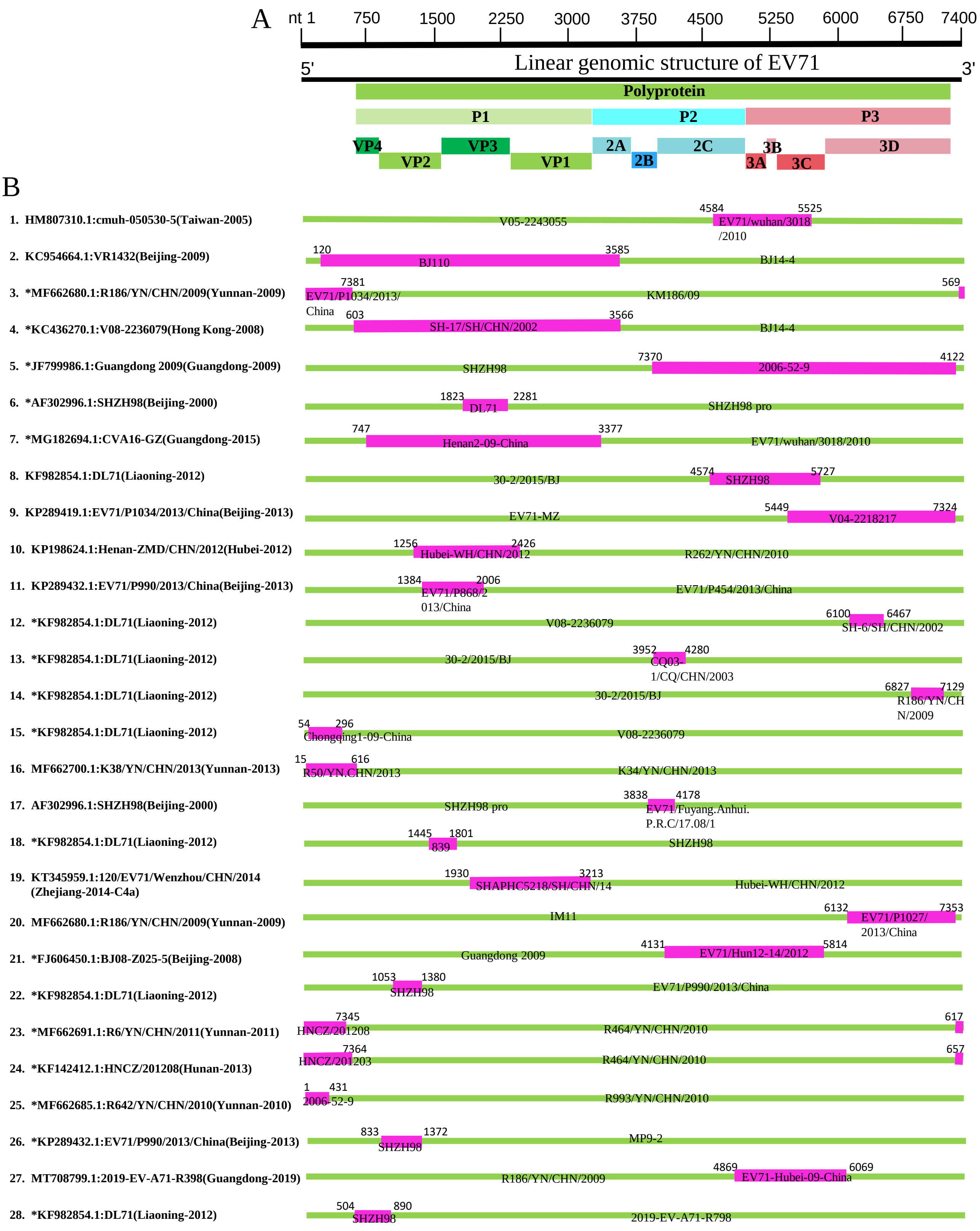

Supplementary Figure S4. Phylogenetic trees based on two different EV71-RNA genomic regions. (**A**) nt 1200-2400. (**B**) nt 4500-5500. The viruses involved in the recombination events 1, 2, 5, 10, and 27 were analyzed using phylogenetic trees to further confirm the recombination events. The trees were constructed using the neighbor-joining methods with 1000 bootstrap replication. The recombinants, minor and major parents are indicated in red, blue, and yellow colors, respectively.

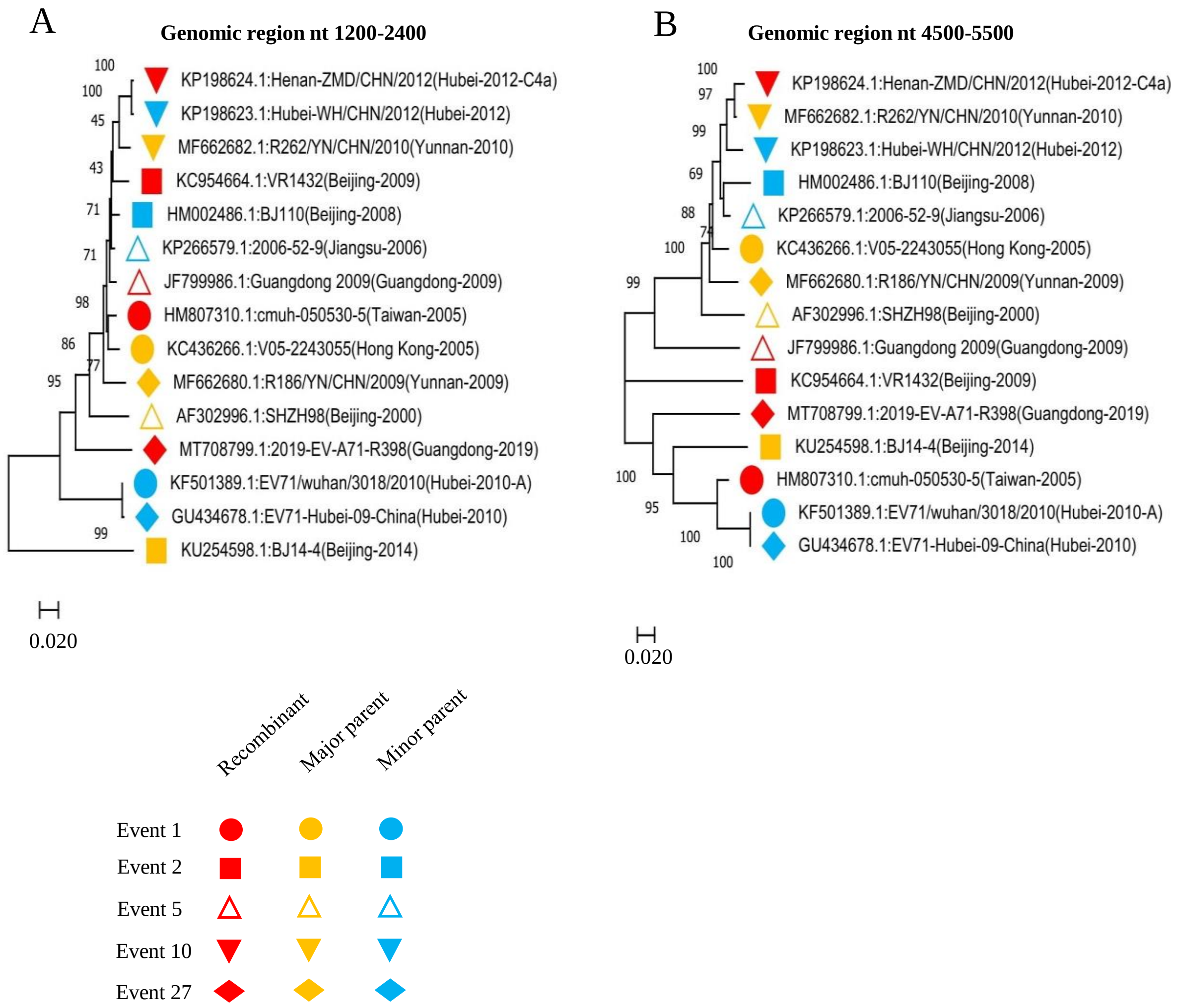

Supplementary Figure S5. Amino acid variability landscape of full-length EV71 polyproteins in China, 1998-2019. The plot represents amino acid variations in aa 1-750 position (**A**), aa 751-1501 (**B**), and aa 1501-2151 (**C**). The ORF nucleotide sequences were used to acquire their consensus amino acid sequence using the Wu-Kabat variability coefficient implemented by PVS. Y-axes represent the Wu-Kabat variability coefficient values, where the estimation limit is 1. Above the limit of 1 represents variations. X-axes represent the amino acid positions. The variability coefficient was calculated as following equation: variability = n*k/N. The n denotes the number of sequences in the alignment, k denotes the number of different amino acids at a given position, and N is the time available for the most frequently identified amino acid.

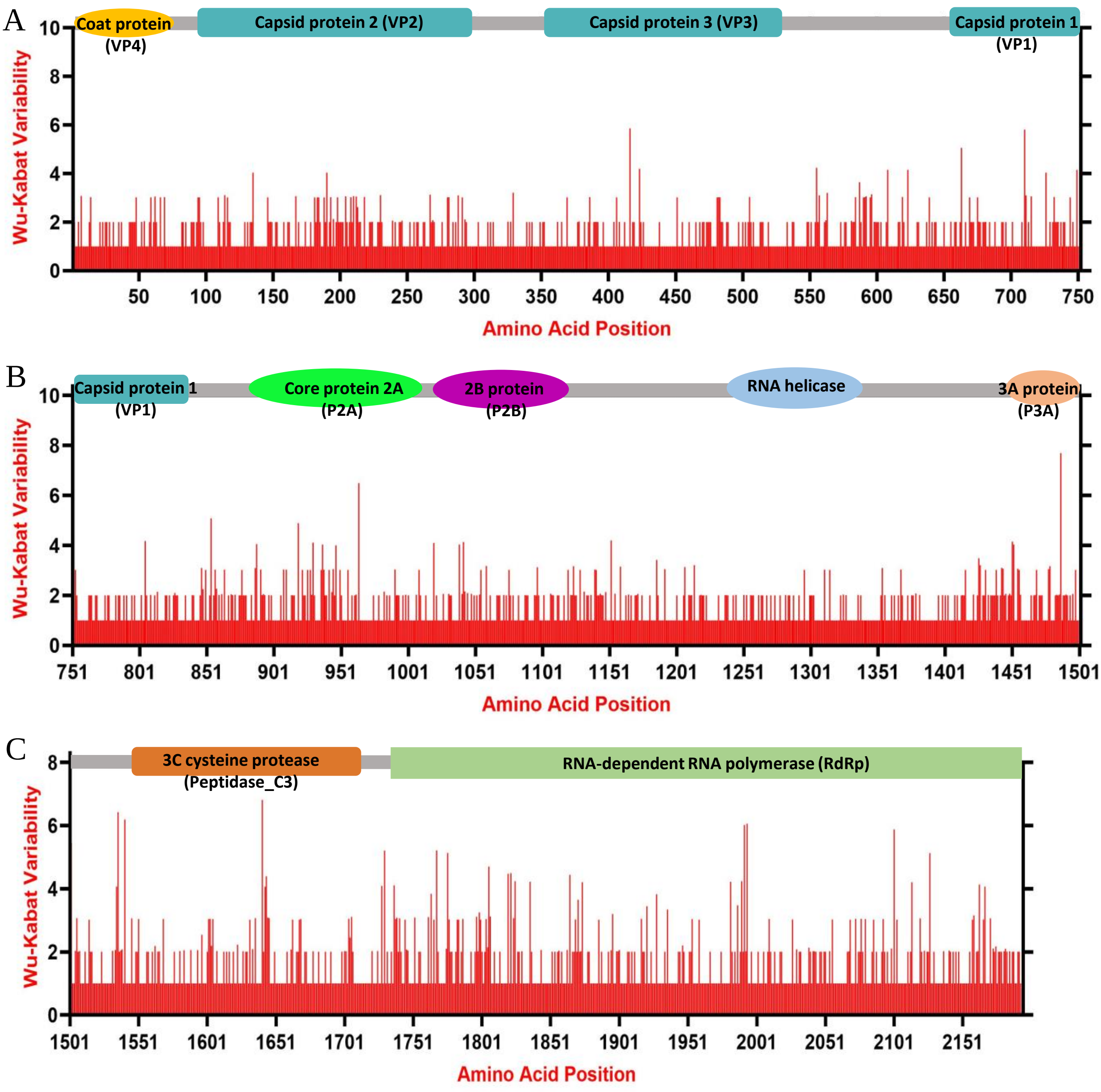

Supplement: Supplementary file 2 [file Data_Sheet_2.PDF]
